# In Vitro Comparison of Compressive and Tensile Strengths ofAcrylic Resins Reinforced by Silver Nanoparticles at 2% and0.2% Concentrations

**DOI:** 10.5681/joddd.2014.037

**Published:** 2014-12-03

**Authors:** Tahereh Ghaffari, Fahimeh Hamedirad, Baharak Ezzati

**Affiliations:** ^1^Dental and Periodontal Research Center, Tabriz University of Medical Sciences, Tabriz, Iran; ^2^Assistant Professor, Department of Prosthodontics, Faculty of Dentistry, Tabriz University of Medical Sciences, Tabriz, Iran; ^3^Post-graduate Student, Department of Prosthodontics, Faculty of Dentistry, Tabriz University of Medical Sciences, Tabriz, Iran

**Keywords:** Polymethyl methacrylate, compressive strength, tensile strength, acrylic resin, silver nanoparticles

## Abstract

***Background and aims.*** Polymethyl methacrylate, PMMA, is widely used in prosthodontics for fabrication of removable prostheses. This study was undertaken to investigate the effect of adding silver nanoparticles (AgNPs) to PMMA at 2% and 0.2% concentrations on compressive and tensile strengths of PMMA.

***Materials and methods.*** The silver nanoparticles were mixed with heat-cured acrylic resin in an amalgamator in two groups at 0.2 and 2 wt% of AgNPs. Eighteen 2×20×200-mm samples were prepared for tensile strength test, 12 samples containing silver nanoparticle and 6 samples for the control group. Another 18 cylindrical 25×38-mm samples were prepared for compressive strength test. Scanning electron microscopy was used to verify homogeneous distribution of particles. The powder was manually mixed with a resin monomer and then the mixture was properly blended. Before curing, the paste was packed into steel molds. After curing, the specimens were removed from the molds. One-way ANOVA was used for statistical analysis, followed by multiple comparison test (Scheffé’s test).

***Results.*** This study showed that the mean compressive strength of PMMA reinforced with AgNPs was significantly higher than that of the unmodified PMMA (P<0.05). It was not statistically different between the two groups reinforced with AgNPs. The tensile strength was not significantly different between the 0.2% group and unmodified PMMA and it de-creased significantly after incorporation of 2% AgNPs (P<0.05).

***Conclusion.*** Based on the results and the desirable effect of nanoparticles of silver on improvement of compressive strength of PMMA, use of this material with proper concentration in the palatal area of maxillary acrylic resin dentures is recommended.

## Introduction

Denture bases can be fabricated using various materials, including metals and heat-cured acrylic resins. Metal denture base is not preferred due to several disadvantages, including lack of retention because of heavy denture, poor esthetic features, cost, difficulty in tissue replacement in severely resorbed alveolar ridge and inability to reline.^1^ Acrylic resins have been used widely because of their good esthetics and favorable characteristics such as easy handling and biocompatibility.^2^ These materials account for approximately 95% of the denture base materials used in prosthodontics. The majority of prosthetic acrylic resins consist of PMMA, polyethyl methacrylate (PEMA), and additional copolymers. Polymethyl methacrylate, PMMA, is commonly used in dentistry for different purposes such as trial base plates, orthodontic functional appliances and denture bases.^3^

PMMA is the most popular denture base material currently available.^4^ Almost all the dentures are fabricated with this type of polymer.^4^ Although the characteristics of this material are not ideal in every aspect, it has many desirable features that make it very favorable. Acrylic resins have excellent esthetic properties, sufficient strength, low water sorption, low solubility, and biocompatibility. They are very accurate in reproducing surface detail and can be easily repaired. Nevertheless, few but important drawbacks are inherent in this resin, such as low thermal conductivity, high coefficient of thermal expansion that causes internal stresses to be released during the process resulting in dimensional inaccuracy and relatively low modulus of elasticity which causes its rapid deformation at low stresses.^4^ As stated, acrylic resins have been successful as denture base because of their ease of processing, low cost, low weight and color matching ability,^5^ but acrylic resins are considerably brittle.^6,7^

Many efforts have been made to overcome its drawbacks. Chemical modification of internal structure of polymer can be used to improve its properties.^8^

PMMA can be reinforced with various types of fibers and fillers to improve its properties.^9,10^ Carbon fibers have been added to dental acrylic resin to improve its fatigue behavior and impact strength.^11^ Furthermore, polyethylene fibers have been suggested to enhance the physical properties of acrylic resins.^12,13^ Reinforcement with metal fillers such as silver and aluminum powder improved some physical and mechanical properties of acrylic resin while addition of silver, copper, and/or aluminum in the form of powder to the resin improved its thermal conductivity, polymerization shrinkage and water sorption.^14^

The antimicrobial properties of silver, especially when the nanoparticles are added to the denture have been reported in several studies.^15-17^ Chladek and colleagues showed that silver nanoparticles inhibit the fungus *Candida albicans* growth and adding this material to the dentures could reduce oral diseases among edentulous patients.^15^ The antibacterial effect of silver is even more noticeable when used as nano-particles.^17^

Silver particles have been used as an addition to acrylic resin in order to improve its mechanical properties.^3,18^ Although adding 25% silver powder to denture base increases its thermal conductivity more than 4 times, it results in a significant decrease in the mechanical properties of acrylic resin, making denture more susceptible to breaking by an impact.^14^

In the past, micrometer-sized particles were used to improve the resin characteristics; however, these particles presented several drawbacks. Regarding advances in nanotechnology sciences and benefits of adding silver nanoparticles to the acrylic base, which leads to better processing and smoother surface compared to micrometer-sized silver powder, the use of silver nanoparticles is preferred. Among various nanofillers available the silver nanoparticles are the most widely used nanoparticles because of their ductility, electrical and thermal conductivity and antimicrobial activity.^19-22^ On the other hand, resin discoloration and high cost can limit its use. The present study was conducted to evaluate the effect of the addition of silver nanoparticles at 2 and 0.2 wt% concentrations on compressive and tensile strengths of acrylic base.

## Materials and Methods

In this research polymethyl methacrylate (PMMA; SR Triplex Hot, Ivoclar Vivadent, Liechtenstein, Germany) as a heat-curing acrylic resin and silver nanoparticles (AgNPs) with a diameter of <35 nm (Model number: SP – A00601, Top Nano Technology Co., Ltd., Iran) were used. AgNPs in two concentration groups at 0.2 and 2 wt% were mixed with heat-curing acrylic resin. These two concentrations were determined based on a pilot study which revealed that addition of less than 2% AgNPs to the acrylic resin will increase compressive strength with no decrease in tensile strength. For best distribution, the mixing procedure was carried out in an amalgamator (Dentine, Esfahan, Iran) for 20 minutes. Mixing of AgNPs was manually carried out with a resin monomer and then the mixture was thoroughly blended. After complete mixing before curing, the paste was packed into steel molds and then the specimens were removed from the molds after curing. All the samples were polished to 400-grit emery paper (grades 320, 500, 800, Nippon Coated Abrasive, Aichi, Japan) to remove excess acrylic resin.

Sample size was determined according to pilot study results and 18 specimens for each test (compressive and tensile strength) and a total of 36 specimens were prepared. Specimens of each test were divided into 3 groups as follows:

Group A: 6 specimens of pure acrylic resin were used as the control group.

Group B: 6 specimens of PMMA were mixed with 0.2 wt% of AgNPs.

Group C: 6 specimens of PMMA were mixed with 2 wt% of AgNPs.

Compressive strength measurement apparatus (100 (kN) cell load capacity, Alfred J Amsler & Co, Germany) and universal tensile strength measurement apparatus (20 (kN) cell load capacity, Zwick Z100, Germany) were used to determine compressive and tensile strengths of the samples. Based on ASTM D 695-02a (ISO 604) standard recommended by the measurement device manufacturer, eighteen compressive strength test samples were prepared. The specimens were formed in cylinders with dimensions of 25×38 mm with a metal mold. Another eighteen specimens were prepared for tensile test with rectangular cubic shape, measuring 2×20×200 mm in size according ASTM D638-10 (ISO 527) recommended by the manufacturer. All the samples were measured by a digital caliper (Guanglu, Strikhlu, Germany) and an error of ±0.03 mm was considered insignificant.

**Figure 1.**
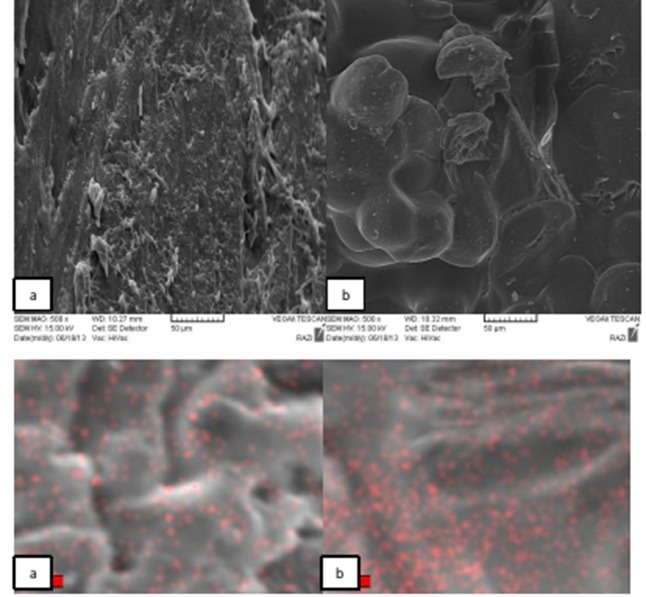
Scanning electron micrograph and Ag map of: a) 0.2% AgNPs; b) 2% AgNPs.

Scanning electron microscopy (SEM, VEGA/TESCAN, Czech Republic) was used to study distribution of nanoparticles and the cross-sectional morphology of the samples (Figure 1).

The specimens were conditioned in the standard laboratory environment for 24 hours before performing the tests (temperature=23±2°C, humidity=50±5%).

Compressive strength test samples were placed in the measuring apparatus in an appropriate manner and cross-sectional area of each sample (mm^2^) was determined. A compressive load (N) was applied at a crosshead speed of 1.3 mm/min. The compressive strength (MPa) was measured at the sample fracture point.

Tensile strength test samples were fixated in the measuring apparatus and cross-sectional area of each sample (mm^2^) was described. A tensile load (N) was applied at a crosshead speed of 5 mm/min. The maximum tensile strength (MPa) was measured at the sample fracture point.

The strengths were measured according to the following formula:

Strength (MPa) =load (N)/cross sectional area (mm^2^)

Mean, average, and mode in each group were calculated and normal distribution curve was evaluated. One-way ANOVA, followed by multiple comparison test (Scheffé’s test), was used for statistical analysis. Statistical significance was set at P<0.05.

## Results

The morphology of the samples in cross-section and Ag map of two samples with different contents of AgNPs are shown in Figures 1aand1b As shown, the sample with 0.2 wt% of AgNPs has a better distribution compared to the sample with 2 wt% of AgNPs. These figures show that an increase in AgNPs caused aggregation of nanoparticles in the sample by extra content, resulting in changes in the fractured surface.

**Figure 2.**
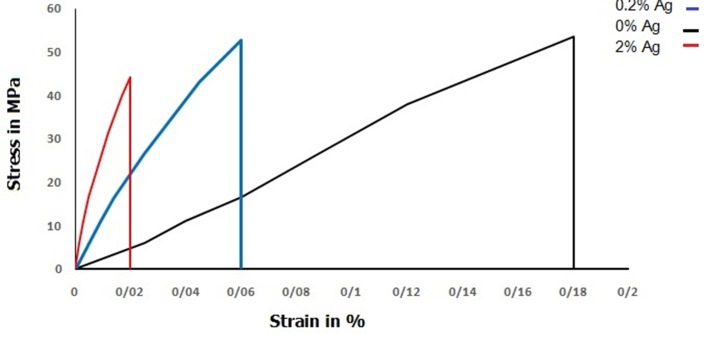
Comparison of compressive strengths of specimens.

One-way ANOVA results presented in Table 1 illustrate significant differences in compressive strength of the groups. Multiple comparison test (Scheffé’s test) results for compressive strengths in various groups showed that acrylic resin at 0.2% and 2% AgNPs concentrations had a significantly higher compressive strength compared with the control group (P<0.05), but the strength difference between the groups containing 0.2% and 2% AgNPs was not significant (P>0.05). The comparisons of compressive strength results of all the groups are shown in Figure 2.

**Table 1:** Means and standard deviations of compressive strengths for the groups tested (in MPa)

			95% confidence interval for mean				ANOVA	
Group	N	Mean ± SD	Lower bound	Upper bound	Maximum	Minimum	f	P
A (control)	6	99.5 ± 3.44	95.879	103.120	95.00	105.00	14.32	.001
B (0.2%)	6	114 ± 5.95	106.601	121.398	104.0	119.00		
C (2%)	6	118.4 ± 10.29	107.733	129.066	104.0	125.00		

**Figure 3.**
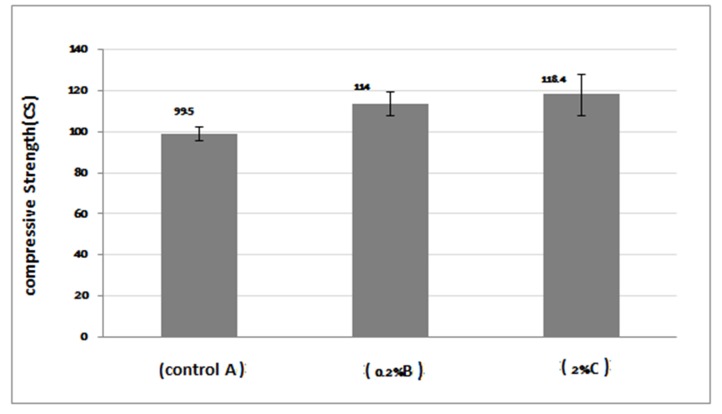
Comparison of tensile strengths of specimens.

The mean, standard deviation, and minimum and maximum stress values of tensile strengths for each experimental group are presented in Table 2. One-way ANOVA revealed a statistically significant difference between the mean values (P<0.05). Scheffé’s test showed no significant differences between the test group with 0.2% AgNPs and the control group (P>0.05). On the other hand, there was a statistically significant difference between the test group with 2% AgNPs and the control group (P<0.05). In other words, the tensile strength significantly decreased in 2% AgNPs test group in comparison with the control group. There were no significant differences between the test groups (P>0.05). The comparisons of tensile strength results of all the groups are shown in Figure 3.

**Table 2:** Means and standard deviations of tensile strengths for the groups tested (in MPa)

			95% confidence interval for mean				ANOVA	
Group	NN	NMean ± SD	Lower bound	Upper bound	Minimum	Maximum	f	P
A (control)	6	48.41 ± 4.85	43.318	53.518	39.62	53.71	6.181	.011
B (0.2%)	6	41.31 ± 6.88	34.085	48.534	31.95	52.64		
C (2%)	6	37.12 ± 4.89	31.983	42.259	30.82	44.29		

**Figure 4.**
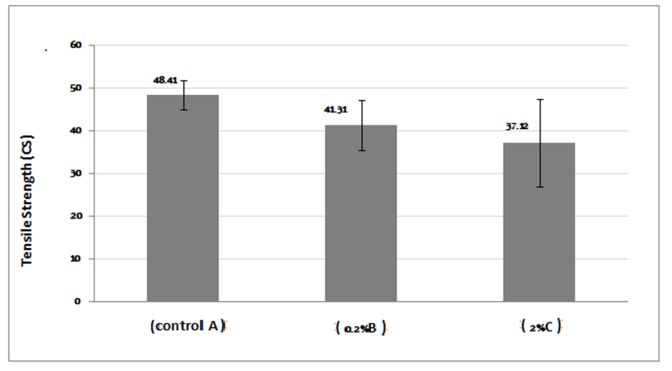
Stress/strain diagram of samples at maximum value.

Figure 4 shows stress/strain diagram of tested samples for various groups at maximum value. The results showed that adding AgNPs to acrylic resin decreased elongation of samples and reduced modulus of toughness. Pure acrylic resins have the highest modulus of elasticity (tangent of curve in the linear portion) and fracture toughness (area under the curve).

## Discussion

This study was conducted to test the effects of AgNPs on mechanical properties of the final acrylic product. In this study two types of mechanical properties, i.e. compressive and tensile strengths of acrylic resin, were studied. In recent years AgNPs have been largely investigated because of their antimicrobial activity. In particular, AgNPs are now considered antibacterial agents due to inhibition of oral pathogens and have been used in various applications.^20,23^ There are many reports about dependence of acrylic resin’s properties on nanoparticle concentrations.^24-29^ Selection of silver as filler in this study was based on properties of this filler. The first reason is the high thermal conductivity of silver, which can improve the thermal conductivity of the denture. In addition, it has been demonstrated that silver not only has no adverse effects in the oral cavity,^30^ but also it can reduce the adhesion *Candidate albicans* and has anti-microbial effects.^15-17^ In addition, some studies have revealed that AgNPS can improve the mechanical properties of acrylic resin.^3,18^ Low concentration of silver would reduce material costs and less monomer would be needed while mixing with acrylic powder. Therefore, the mechanical properties of the final polymer would not be compromised.^31,32^Chladek et al reported that the mechanical and physical properties of the composite are influenced by silver nanoparticle concentration. They also showed that mechanical properties of composites decreased by increasing silver nanoparticles.^18^ It has been demonstrated that addition of more than 5 wt% of the metal fillers into acrylic resin would reduce tensile strength.^14^

According to the results, AgNPs with 0.2 and 2 wt% increased the compressive strength of acrylic resins, but increasing AgNPs concentration from 0.2 wt% to 2 wt% did not improve compressive strength of acrylic resin significantly. The acrylic resin is a brittle material but at compressive conditions behaves like ductile materials.^33^

Tensile strength results showed different behavior patterns and incorporation of AgNPs decreased tensile strength in both groups containing AgNPs. Decrease in tensile strength was not significant by addition of 0.2 wt% AgNPs, whereas high concentrations of 2 wt% AgNPs exhibited significantly low tensile strength. These findings are consistent with previous studies.^3,18^ AgNPs acts as impurities and tensile strength would decrease by increasing their content. Dispersion of nanoparticles in acrylic PMMA matrix decreases the reaction of monomers and increases the amount of unreacted monomer, behaving like a plasticizer.^29^ This shows the importance of the additive content of nanoparticles.

As the Ag mapping results showed increasing AgNPs content resulted in agglomeration of these nanoparticles, increasing impurity action of nanoparticles. Impurities or particulate agglomerate compounds act as stress concentration centers in the matrix and unfavorably decrease mechanical properties of resin.^24^

According to the results of modulus of toughness in Figure 10, the acrylic resin with AgNPs is more brittle than pure resin because of low energy absorption during fracture. Dysfunctional effect of high concentrations of AgNPs can be confirmed by these results and also brittleness of nanocomposite samples may confirm the high compressive strength of these samples because brittle materials have high compressive strength and low tensile strength.^34^ It can be concluded from these findings that AgNPs in high concentration result in agglomeration sites, which function as impurities and decrease tensile strength.

Another disadvantage of metallic fillers in acrylic resin is color change which is important in esthetic areas. AgNPs also caused some black discoloration in the acrylic resin, limiting its application in the appearance zone. However, we can apply low concentrations of silver nanoparticles to the palatal area of the denture, which is exposed to the highest stresses of mastication.

The results of this article were based on an “in vitro” study; so future “in vivo” studies can be conducted to evaluate the effects of these changes in dentures on clinical performance and patient satisfaction.

## Conclusion

This study was conducted to evaluate the effect of adding AgNPs to PMMA with two different weight percentages on two properties of acrylic resin.

The two tested properties were compressive and tensile strengths. The results showed that the effect of AgNPs significantly depends on its concentration. Based on the results adding AgNPs with proper concentrations to PMMA can improve its mechanical characteristics without any adverse effects and is strongly recommended in the palatal portion of acrylic base of complete maxillary dentures.

Because adding AgNPs by 2 wt% decreased the tensile strength of acrylic resin, this study was performed on different mixtures with various percentages of AgNPs in order to determine the favorite weight percentage which is not associated with this disadvantage.
